# *Eimeria*: Navigating complex intestinal ecosystems

**DOI:** 10.1371/journal.ppat.1012689

**Published:** 2024-11-22

**Authors:** Shengjie Weng, Erjie Tian, Meng Gao, Siyu Zhang, Guodong Yang, Bianhua Zhou

**Affiliations:** College of Animal Science and Technology, Henan University of Science and Technology, Luoyang, Henan, People’s Republic of China; Joan and Sanford I Weill Medical College of Cornell University, UNITED STATES OF AMERICA

## Abstract

*Eimeria* is an intracellular obligate apicomplexan parasite that parasitizes the intestinal epithelial cells of livestock and poultry, exhibiting strong host and tissue tropism. Parasite–host interactions involve complex networks and vary as the parasites develop in the host. However, understanding the underlying mechanisms remains a challenge. Acknowledging the lack of studies on *Eimeria* invasion mechanism, we described the possible invasion process through comparative analysis with other apicomplexan parasites and explored the fact that parasite–host interactions serve as a prerequisite for successful recognition, penetration of the intestinal mechanical barrier, and completion of the invasion. Although it is recognized that microbiota can enhance the host immune capacity to resist *Eimeria* invasion, changes in the microenvironment can, in turn, contribute to *Eimeria* invasion and may be associated with reduced immune capacity. We also discuss the immune evasion strategies of *Eimeria*, emphasizing that the host employs sophisticated immune regulatory mechanisms to suppress immune evasion by parasites, thereby sustaining a balanced immune response. This review aims to deepen our understanding of *Eimeria*–host interactions, providing a theoretical basis for the study of the pathogenicity of *Eimeria* and the development of novel anticoccidial drugs.

## Coccidiosis and intestinal susceptibility

Coccidiosis, a significant intestinal parasitic disease mainly caused by *Eimeria* spp., leads to an estimated $13 billion in economic losses to the global poultry industry annually, around $140 million to small ruminants, and approximately $723 million to cattle [1,2]. Although *Eimeria* spp. can infect most animal species, different *Eimeria* spp. exhibit high host specificity [3]. Furthermore, parasites within the same genus, *Eimeria*, demonstrate different tissue tropism, as evidenced by chicken *Eimeria* spp., which specifically infect different regions of the intestine. Understanding the general mechanisms governing parasitic tropisms has long been challenging.

A sporulated oocyst contains 4 sporocysts, each containing 2 sporozoites [4]. Under suitable host conditions, the 8 sporozoites are released from the sporocysts [5]. The molecular and biochemical mechanisms underlying decapsulation are not well understood; however, it is currently hypothesized that host environmental factors such as temperature, pH, proteases, bile salts, and other unidentified factors may trigger the decapsulation of ingested sporulated oocysts (**Box 1**). To fully comprehend the specificity of *Eimeria* parasitism, it is necessary to consider the following factors: (i) recognition by intestinal receptors; (ii) intestinal mechanical barrier; (iii) intestinal microbiota; and (iv) intestinal immunity.

## Target cell recognition and invasion

The released sporozoites serve as the primary infection unit of the host, initiating the recognition of the target cell. Although host cells do not actively facilitate physical parasite entry into the cell, they may provide surface molecules, receptors, or secreted metabolites that attract or activate apicomplexan parasites [6]. Several studies have shown that this facilitation may be influenced by the unique conditions of the intestinal cavity, including factors such as pH, enzymes, mucus, metabolites, nutrient concentrations, and microorganisms [7–9].

### Recognition and adhesion of the target cell

There are 2 hypotheses regarding the mechanism of target cell recognition by Apicomplexa. Apicomplexan parasites, such as *Toxoplasma gondii* (*T*. *gondii*), *Eimeria*, and *Cryptosporidium*, have abilities to invade various host cells [10]. Several studies have shown that *Eimeria tenella* (*E*. *tenella*) sporozoites are able to invade Madin–Darby bovine kidney (MDBK) cells and produce mature schizonts and first-generation merozoites [11]. When infected with second-generation merozoites from wild-type strains, gametes, and oocysts of *E*. *tenella* were observed in a chicken lung epithelial cell line [12]. These studies suggest that infection with *Eimeria* is neither host- nor cell type-specific, since many cell types can be infected by sporozoites or merozoites. The first hypothesis is that the invasion process of Apicomplexa is mostly independent of host receptors but rather relies on the formation of “moving junction (MJ)” to create their own anchors [13,14]. MJ is also considered significant markers during the invasion of Apicomplexa [15]. However, different *Eimeria* species infect different regions of the intestine. *E*. *acervulina* and *E*. *praecox* parasitize the duodenum and jejunum, whereas *E*. *maxima* parasitizes the jejunum and ileum, *E*. *necatrix* develops in the jejunum, ileum, and cecum. Conversely, *E*. *mitis* infects cells of the ileum, *E*. *brunetti* parasitizes the cecum and rectum, and *E*. *tenella* only parasitizes the cecum [16–19]. This suggests that different regions of the intestine may possess specific receptor molecules involved in *Eimeria* invasion. Consequently, a second hypothesis, known as the receptor hypothesis, appears more plausible, proposing that the presence of specific receptors in host cells promote the recognition of surface ligands *on Eimeria* [20]. Further evidence points to the existence of *Eimeria* ligands that bind to surface molecules of intestinal epithelial cells (IECs), including antigens of 18–19, 30, and 45–60 kDa, as well as membrane glycoconjugates and other molecules [21–23]. For the invasion of *E*. *tenella* sporozoites in vitro, D-galactose residues of the sporozoite surface and D-galactose recognizing receptors of the host cells are important factors [23]. Several studies have shown that the binding of monoclonal antibodies to the *E*. *tenella* sporozoites surface antigen can significantly inhibit the penetration to host cells [21,24,25]. However, inhibiting the binding of these ligands to receptors does not completely prevent invasion, suggesting the existence of other invasion mechanisms or alternative recognizable receptors [6].

Over the past 2 decades, research has revealed the involvement of microneme proteins in cell recognition and adhesion, with increasing evidence of their association with host and tissue tropism [26]. Among the *Eimeria* spp., the invasion mechanism of *E*. *tenella*-induced by microneme proteins has been extensively studied, and 11 microneme proteins of *E*. *tenella* have been characterized, including *Et*MIC1 to 5, *Et*MIC 7 to 8, *Et*MIC13 and apical membrane antigen 1 to 3 (*Et*AMA1-3) [27,28]. These *Et*MICs contain an adhesion domain that binds to the oligosaccharide epitopes of host cells (Table 1). The adhesion domain, known as microneme adhesive repeat domains (MARs), is exclusively conserved among apicomplexan parasites [29]. All apicomplexan MARs were classified into Types I and II domains based on their subtle structural differences, and *T*. *gondii* and *Plasmodium* possess Types I and II MARs. However, *Eimeria* parasites only possess only Types I MAR domain, which may be related to the host and tissue tropism [9,30]. Within the MIC family of *Eimeria*, MIC3 has garnered the most extensive attention and appears to be critical in determining tissue tropism [9,31,32]. Although *T*. *gondii* MIC1 (*Tg*MIC1), *Tg*MIC13, *Neospora caninum* MIC1 (*Nc*MIC1), and *Et*MIC3 can recognize and bind to sialic acid on the surface of IECs, the 4 MICs also display differences in binding preferences, which may be related to the different MARs of MICs [8,9,29]. Several studies have shown that *Et*MIC3 MAR1b lacks recognition of NeuGc-terminating glycans, a type of salivary acid, which are rare in the chicken host [9,33]. These differing specificities of salivary acid recognition are clearly major factors in the host tropisms of *Eimeria*. Additional research indicates that the cecum contains more N-glycoconjugates than the duodenum, jejunum, and ileum [34]. Interestingly, preferred binding site of *Et*MIC3 is in the cecum that expresses a high level of α2–3 sialylated glycans, which is a key factor in guiding *E*. *tenella* to the site of invasion in the cecum [9]. Notably, when *Ea*MIC3-*Et*MIC3 with the *Et*MIC3-MAR1b domain was constructed, it exhibited strong binding to the cecum but lost the ability to interact with the duodenum and jejunum [30]. Upon comparing the binding abilities of *Et*MIC1, *Et*MIC2, *Et*MIC3, and *Et*AMA1 to various regions of the chicken intestine, it was discovered that only *Et*MIC3 exhibited binding capability to the cecum, whereas *Et*MIC1, *Et*MIC2, and *Et*AMA1 did not bind to any regions of the chicken intestine [35]. Most recently, a novel protein identified as *Et*MIC8, showed 95.63% homology with *E*. *necatrix*, which may be related to the common parasitic characteristic of these 2 *Eimeria* species in the cecum [28]. Additionally, a chicken epithelial cell adhesion molecule (EPCAM) has been identified that can bind to the epidermal growth factor-like domain (EGF) of *Et*MIC8 (*Et*MIC8-EGF). Furthermore, an EPCAM-specific antibody inhibited the binding of *Et*MIC8-EGF to IECs in a concentration-dependent manner [36]. In future studies, exploring the specific binding of glycoconjugates and *Eimeria* MICs at the molecular level could be the key to understanding tissue tropism.

**Table 1:** Specific *Et*MICs, host receptor, and functions of *Et*MICs.

*Et*MICs	Host receptors	Functions	References
*Et*MIC1	unknown	Adhesion, invasion, the development of pv	[37–39]
*Et*MIC2	unknown	Form a complex with *Et*MIC1, adhesion, invasion;	[37,40,41]
*Et*MIC3	BCL2-associated athanogene 1 (BAG1), Endonuclease polyU-specific-like (ENDOUL), α2–3 sialylated glycans	Inhibits apoptosis of host cells recognition and adhesion	[9,35]
*Et*MIC4	epidermal growth factor receptor (EGFR), thrombospondin type-1	Recognition, adhesion inhibits apoptosis of host cells	[42–45]
*Et*MIC5	unknown	Form a complex with *Et*MIC4, recognition, and adhesion	[43,46]
*Et*MIC7	EGFR	Inhibits apoptosis of host cells	[47]
*Et*MIC8	epithelial cell adhesion molecule (EPCAM)	Recognition, adhesion invasion	[28,36]
*Et*MIC13	unknown	unknown	-

### The process of invasion

Apicomplexan parasites initiate invasion upon specific recognition of target cells, a process that relies on motility and anchoring to host cells [13]. Anchoring is a conserved process that involves the secretion of proteins by the microneme and rhoptry organelles through exocytosis [48]. The rhoptry, a key organelle of apicomplexan parasites, secretes rhoptry bulb proteins (ROPs), which play crucial roles in invasion, parasitophorous vacuole (PV) formation, and host cell regulation [49]. Rhoptry neck protein 2 (RON2), a transmembrane protein secreted by the rhoptry, adheres to and embeds in the host cell membrane. It forms a complex with the microneme secretion protein AMA1, thus establishing and maintaining the integrity of the MJ during invasion [50,51]. Indeed, the indispensable role of the AMA1-RON2 complex in the invasion of *Plasmodium vivax* and *T gondii* has been widely acknowledged [51,52]. However, whether the RONs-AMA1 complex plays a critical role in Apicomplexa invasion remains a topic of intense controversy. Interestingly, RON4 is an essential protein for the construction of the MJ during invasion, functioning independently of AMA1 [53,54]. Similar to RON4, RON5 and RON8 are retained together in the host plasma membrane to assist RON2 in completing the assembly of MJ [52]. Nonetheless, the MJ serves as a robust anchor for the host cell to withstand shear forces during invasion. Subsequently, driven by its own actin motor, the parasite enters the cell interior. The intracellular parasites maintain a living space by secreting dense granule proteins (GRAs) via their dense granules, which, along with the ROPs and MICs, contribute to the formation of the PV [49].

**Fig 1:**
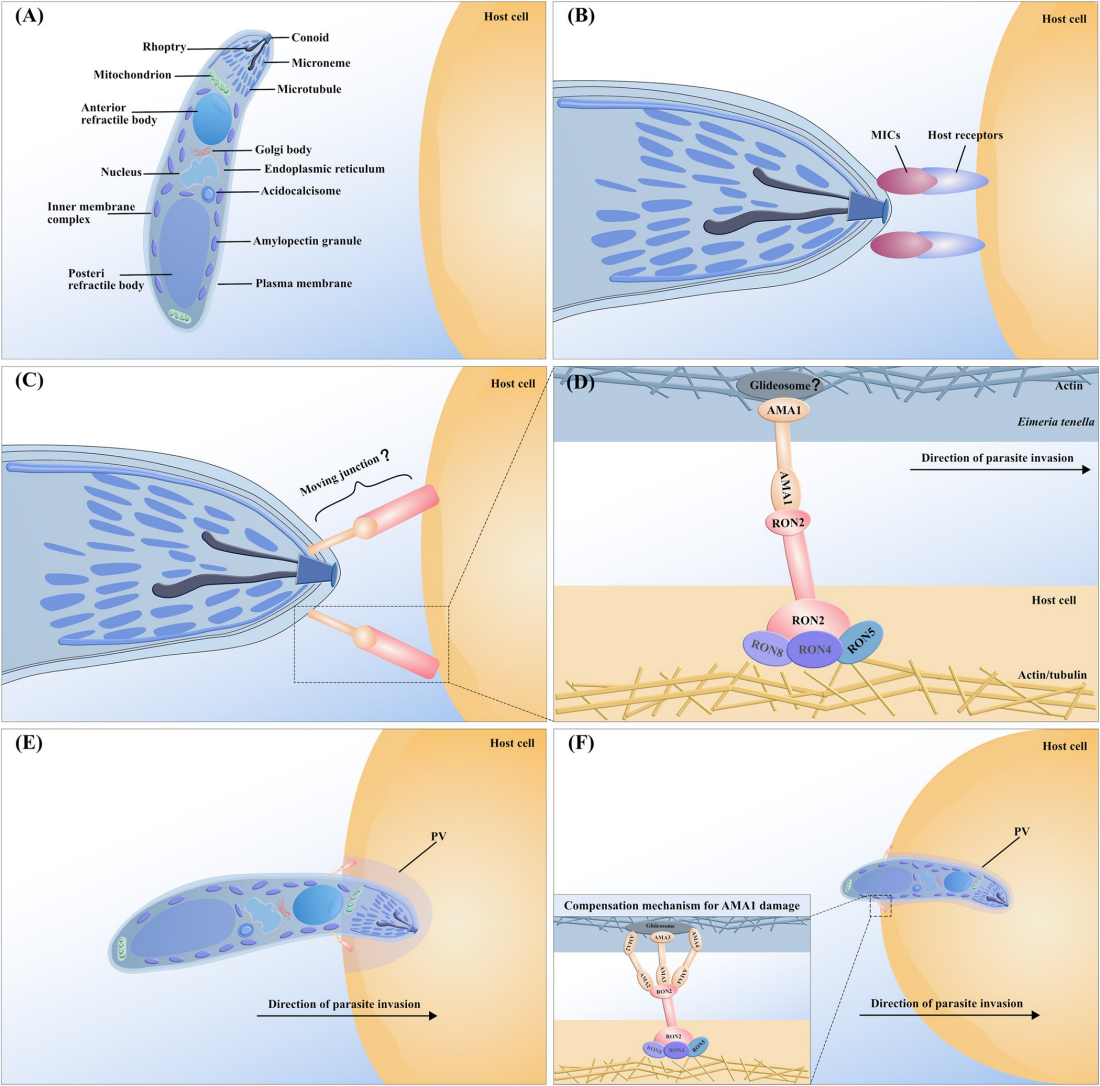
Invasion stages of host cells by *Eimeria*. (A) Cellular structure and organ composition of a generic sporozoite of *Eimeria* species. (B) MICs secreted by *Eimeria* micronemes are involved in the recognition of host cell receptors. (C) After recognition, the released RONs establish complexes with AMA1 to build the MJ. (D) *Eimeria* allows the delivery of the rhoptry neck proteins RON2, RON4, RON5, and RON8 into the host cell and intimate interaction of the extracellular part of the transmembrane protein RON2 with AMA1. On the parasite side, the cytoplasmic tail of AMA1 may interface with the glideosome complex. On the host cell side, actin and tubulin accumulate at the MJ and RONs subvert host protein networks. (E) *Eimeria* maintains living space through the secretion of GRAs from dense granules, which together with the ROPs, and MICs, form the PV. (F) *Eimeria* may deploy 3 additional AMA-RON complexes: AMA2/RON2, AMA3/RON2_L2,_ and AMA4/RON2_L1_ to compensate for the loss of the core AMA1-RON2 complex. The existence of this compensation mechanism, which primarily draws reference from *T*. *gondii*, in *Eimeria* remains unknown at this point. MICs, microneme proteins; RON2, rhoptry neck protein 2; RONs, rhoptry neck proteins; AMA1, apical membrane antigen 1; MJ, moving junction; GRAs, dense granule proteins; ROPs, rhoptry bulb proteins; PV, parasitophorous vacuole.

Regrettably, to date, there remains a dearth of direct studies validating the mechanism of MJ during the invasion process of *Eimeria*. Notably, *Et*AMA1 is prominently expressed at sporozoite apices, and antibodies against *Et*AMA1 or specific *Et*AMA1-binding peptides have been shown to inhibit the invasion of *E*. *tenella* sporozoites [55,56]. However, *Et*AMA1-induced immune protection is limited, possibly linked to *E*. *tenella* genetic and antigenic diversity [57]. An alternative plausible explanation is that *Eimeria* possesses molecular plasticity to cope with unforeseen challenges during invasion, a phenomenon already demonstrated in *T*. *gondii* and *Plasmodium* [52,58]. In the absence of AMA1, *T*. *gondii* can deploy 3 additional AMA-RON complexes: AMA2/RON2, AMA3/RON2_L2,_ and AMA4/RON2_L1_ to compensate for the loss of the core AMA1-RON2 complex [52]. Similarly, such a compensatory mechanism seems to exist in *Eimeria*. Inhibition of *Et*AMA3 expression significantly reduced the invasive ability of *E*. *tenella*. [27]. Additionally, *Et*RON2 has also been demonstrated to possess high immunogenicity, capable of inhibiting sporozoite invasion [59,60]. Moreover, co-immunization with *Et*AMA3 and *Et*RON2_L2_ provided superior immunoprotection compared to single immunization with either *Et*AMA3 or *Et*RON2_L2_ [61]. Multiple studies have indicated that most MJ complex proteins are conserved among Apicomplexa [13,14,62]. Interestingly, *Et*AMA1 exhibits homology with *Tg*AMA1 [56], and *Tg*AMA1 binds to *Et*RON2 with a high affinity to some extent [63]. Although direct evidence for the existence of AMAs-RONs complexes in *Eimeria* spp. is lacking, the inference of their presence based on current research appears reasonable. By drawing upon research from other apicomplexan parasites, we have predicted the invasion process of *Eimeria* (Fig 1).

## Interaction between *Eimeria* and the intestinal mechanical barrier

The tight junctions (TJs) of IECs play a crucial role in maintaining the intestinal barrier against pathogenic microorganisms [64]. After infection with *E*. *maxima*, the instillation of fluorescein isothiocyanate dextran (FITC-D) on day 7 led to the detection of a significant amount of FITC-D in the bloodstream 2 h later, indicating an increase in intestinal permeability (IP) [65]. During *E*. *maxima* invasion, the maturation and release of a large number of second-generation schizonts results in extensive rupture of chicken mid-intestinal epithelial cells, leading to leakage of the midgut intestinal contents [66]. The reduction in tight junction proteins, specifically ZO-1 and occludin, in the cecum of *E*. *tenella*-infected chickens suggested that this leakage may be associated with decreased expression of TJs [67].

The TJs, serving as the gateways between IECs, precisely regulate the efficient passage of nutrients and water, thereby being essential for maintaining nutritional absorption and internal environmental stability. Brush border absorption is largely driven by Na^+^-nutrient cotransporters that rely on the Na^+^ gradient between the intestinal lumen and the cytoplasm of epithelial cells [68]. The permeation through claudin-2 and claudin-15 channels facilitates the movement of Na^+^ from the lamina propria towards the lumen, subsequently enabling further rounds of Na^+^-nutrient cotransport [69,70]. In *Eimeria*-infected chickens, reduced expression of key nutrient transporters, such as Glucose Transporter 2 (GLUT2), GLUT5, and Sodium/Glucose Cotransporter 1 (SGLT1), leads to decreased digestion rates of amino acids, energy, and fats [71–73]. A plausible explanation is that excessive damage to TJs disrupts the paracellular Na^+^ transport pathway, rapidly depleting luminal Na^+^ through transcellular transport and halting nutrient cotransport at the apical brush border membrane [68]. Unfortunately, there is still a lack of research to further elucidate the correlation between tight junctions and Na^+^-glucose cotransport during *Eimeria* infection [71]. Certainly, the overall decline in growth performance is a consequence resulting from the combined impact of reduced expression of nutrient transporter proteins and damage to TJs following *E*. *maxima* infection [71].

*Eimeria* parasites facilitate invasion and colonization by disrupting the TJs of IECs. Using the transmission electron microscopy, it has been observed that second-generation merozoites of *E*. *necatrix* and *E*. *tenella* migrate to host cells located in the lamina propria for development [74]. Claudin proteins, which are integral components of TJs, serve crucial roles in maintaining cell polarity, regulating paracellular permeability, and modulating cell proliferation, transformation, as well as migration processes [75]. Notably, claudin-1 potently inhibits cell migration facilitated by recepteur d’origine nantais [76]. The invasion of *E*. *tenella* and *E*. *maxima* inhibits the expression of claudin-1 [77,78]. Consequently, a possible mechanism is that during invasion, *Eimeria* disrupts TJs between infected and healthy cells, allowing parasites to breach the epithelial barrier and colonize the base of intestinal glands via cell migration [74]. Additionally, apicomplexan parasites possess multiple strategies to penetrate the intestinal epithelial barrier. *T*. *gondii* primarily penetrates the intestinal epithelial barrier into deeper tissues, including the brain, placenta, and retina, through the paracellular pathway [79]. *T*. *gondii* excretes/secretes proteases that disrupt the TJs within IECs, facilitating the paracellular migration of tachyzoites [80]. The leaky epithelium in γδ iIEL-deficient mice was attributed to an absence of occludin phosphorylation and loss of claudin-3 and ZO-1 from the TJ complex. Compared to WT mice, γδ iIEL-deficient mice showed a similar rapid transit of parasites across the intestinal epithelium and increased parasite burden, with the majority of parasites localized to the subepithelial dome [81]. Nevertheless, an alternative perspective suggests that *T*. *gondii* tachyzoites can traverse the host intestinal epithelial barrier by means of the paracellular route through interactions with occludin. Utilizing small interfering RNA (siRNA) to reduce endogenous occludin expression significantly suppresses the transepithelial migration of *T*. *gondii* [82]. Despite the limited insights gained from these few studies into the effects of apicomplexan parasites on TJs, the precise role of the host TJs-associated network of proteins remains inadequately elucidated.

### Indirect effects of molecules released by *Eimeria*

**Fig 2:**
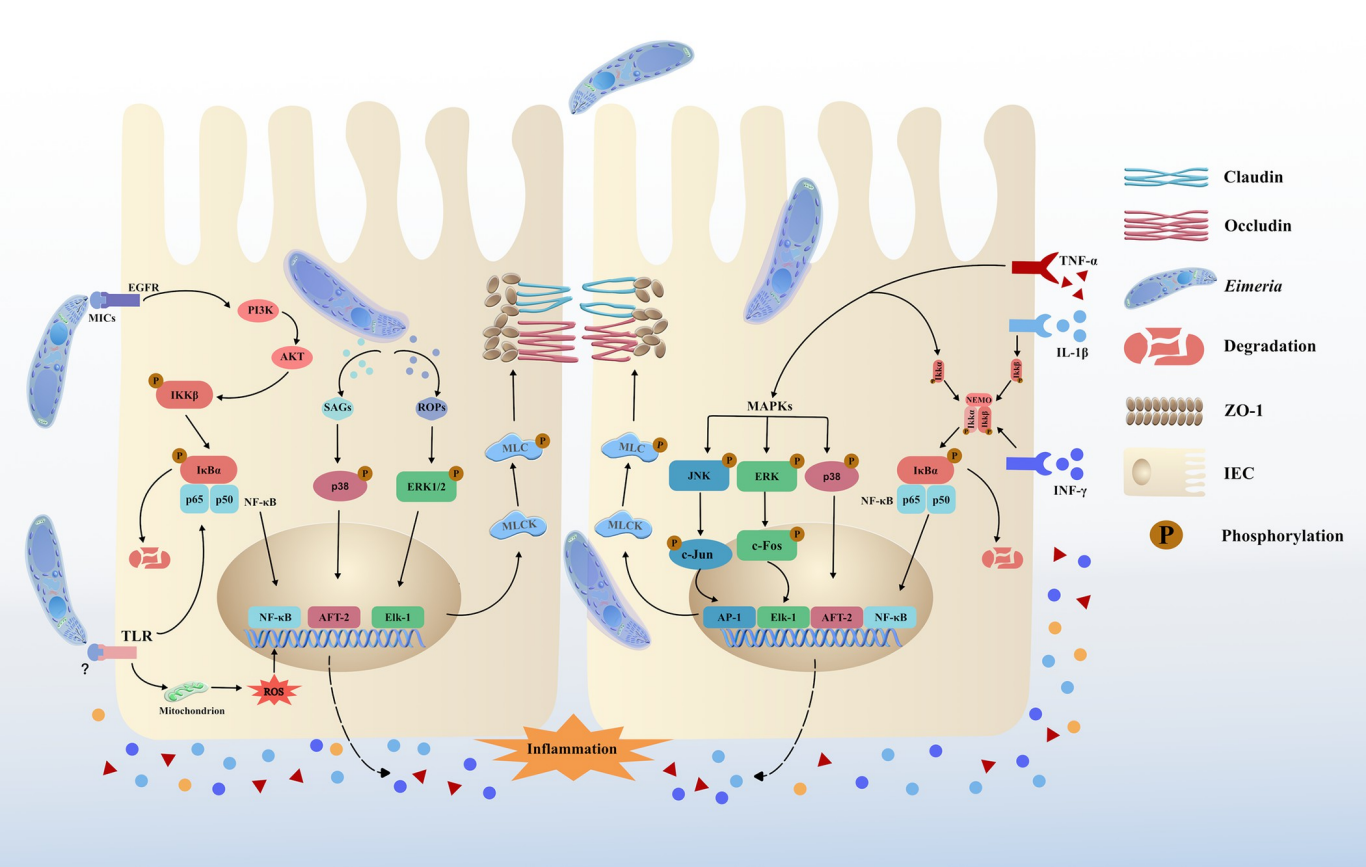
Overview of strategies for breaking through TJs among IECs. *Eimeria* secretes MICs and other molecules that can activate the EGFR-AKT or TLR pathway, leading to the degradation of inhibitor of IκBα and translocation of NF-κB from the cytoplasm to the nucleus. The sporozoites colonized within IECs release surface SAGs and ROPs, which up-regulate the expression of MAPK family members (e.g., p38 MAPK and ERK), thereby accelerating the expression of transcription factors, such as AFT-2 and EIK-1. These combined effects promote the expression of activates intranuclear expression of nuclear NF-κB [100,103] (Fig 2, left). Over-transcription of NF-κB causes the release of inflammatory cytokines, such as interleukin-1β (IL-1β), TNF-α, and interferon-γ (IFN-γ). These Inflammatory cytokines similarly accelerate the degradation of IκBα. NF-κB p65 and p50 are released and translocated into the nucleus. Furthermore, TNF-α activates the MAPKs pathway (p38, JNK, and ERK), which facilitates the phosphorylation of c-Jun, c-Fos, and p38. These phosphorylated proteins ultimately promote the intranuclear expression of nuclear NF-κB by activating AP-1, ELK-1, and ATF-2 [103] (Fig 2, right). Over-transcribed NF-κB ultimately mediates the activation of MLCK-MLC by catalyzing the phosphorylation of MLC (p-MLC). p-MLC eventually induces the opening of TJ, which is mainly regulated by ZO-1, occludin and claudin proteins. EGFR, epidermal growth factor receptor; AKT, serine/threonine kinase; TLR, Toll-like receptor; NF-κB, nuclear factor kappa-B; IECs, intestinal epithelial cells; SAGs, surface antigen proteins; ROPs, rhoptry bulb proteins; MAPK, mitogen-activated protein kinase; ERK, extracellular regulated protein kinase; AFT-2, transcription factor-2; EIK-1, Ets-like protein-1; TNF-α, tumor necrosis factor-α; JNK, c-Jun N-terminal kinase; AP-1, Activator Protein-1; MLCK, myosin light chain kinase; MLC, myosin light chain.

During the early stages of invasion, *Eimeria* parasites secrete MICs that can activate the epidermal growth factor receptor (EGFR)-serine/threonine kinase (AKT) signaling pathway [83]. This activation up-regulates the expression of nuclear factor kappa-B (NF-κB) genes [84]. Additionally, the NF-κB signaling pathway further activates myosin light chain kinase (MLCK)-myosin light chain (MLC) by catalyzing the phosphorylation of MLC (p-MLC) [85,86]. Elevated levels of p-MLC are believed to be a key molecular mechanism underlying inflammation-induced intestinal barrier dysfunction and ultimately increased IP [86,87]. Studies have shown that 4 proteins, namely, *E*. *maxima* surface antigen (*Em*SAG), *Em*HPSP-1, *Em*HPSP-2, and *Em*HPSP-3, can up-regulate the expression of mitogen-activated protein kinase (MAPK) family members (e.g., p38 MAPK, extracellular regulated protein kinase (ERK) and c-Jun N-terminal kinase (JNK)) in host cells during the early stages of invasion [88]. ERK1/2, in particular, can influence the IP by affecting the cytoskeleton or through cascading inflammatory responses [89,90]. A recent study also demonstrated that *Trichinella spiralis* trypsin (*Ts*Tryp) can directly promote the phosphorylation of ERK1/2, contributing to increased IP [91]. In *E*. *tenella*, the *Et*ROP2 protein can activate the p38MAPK and NF-κB signaling pathways, leading to intestinal damage following infection [92,93]. Thus, during the early stages of infection, *Eimeria* parasites exploit their ability to bind to host cells through self-secreted proteins to enhance IP, thereby facilitating early invasion (Fig 2). This demonstrates the cunning nature of *Eimeria* in achieving parasitism.

### Host intestinal inflammation induced by *Eimeria*

*Eimeria* infection triggers the release of various inflammatory cytokines, such as interleukin-1β (IL-1β), tumor necrosis factor-α (TNF-α), and interferon-γ (IFN-γ), by Th1 cells, M1 macrophages, and natural killer (NK) cells [94–97]. These cytokines induce inflammation in IECs, leading to disruption of the intestinal mucosal barrier and decreased expression of tight junction proteins [98]. The production of inflammatory cytokines is partly due to the stimulation of host NF-κB and MAPK by secretory proteins of *Eimeria* and partly due to the innate immune response in the intestine [97]. It has been reported that *Eimeria* sporozoites specifically activate the chicken Toll-like receptor 15 (ChTLR15)/NF-κB/NOD-like receptor thermal protein domain associated protein 3 (NLRP3)/IL-1β pathway, which causes inflammatory damage and TJ disruption in IECs [99]. IL-1β up-regulates the expression of mitogen-activated protein kinase kinase kinase-1 (MEKK-1), leading to the degradation of inhibitor of nuclear factor-κB (IκBα) and the translocation of NF-κB from the cytoplasm to the nucleus [100]. In parallel, this approach activates the classical NF-κB p50/p65 signaling pathway and up-regulates the expression of MLCK, thus disrupting the TJs of IECs [100]. IFN-γ/TNF-α-induced intestinal barrier dysfunction is typically accompanied by increased expression of p-MLC, which is also regulated by NF-κB [85,101,102]. TNF-α can increase the expression of phosphorylated JNK (p-JNK) and p38MAPK, further promoting the intranuclear transcription of NF-κB [103]. Additionally, TNF-α rapidly activates ERK1/2, leading to the phosphorylation and activation of Ets-like protein-1 (Elk-1). Activated Elk-1 binds to serum-responsive elements on the MLCK promoter, resulting in activation of the MLCK gene and MLCK-dependent opening of the TJ [104]. Consequently, the disruption of TJs continues to occur during infection as inflammatory factors are secreted (Fig 2).

## Interactions between *Eimeria* and the gut microbial barrier

**Fig 3:**
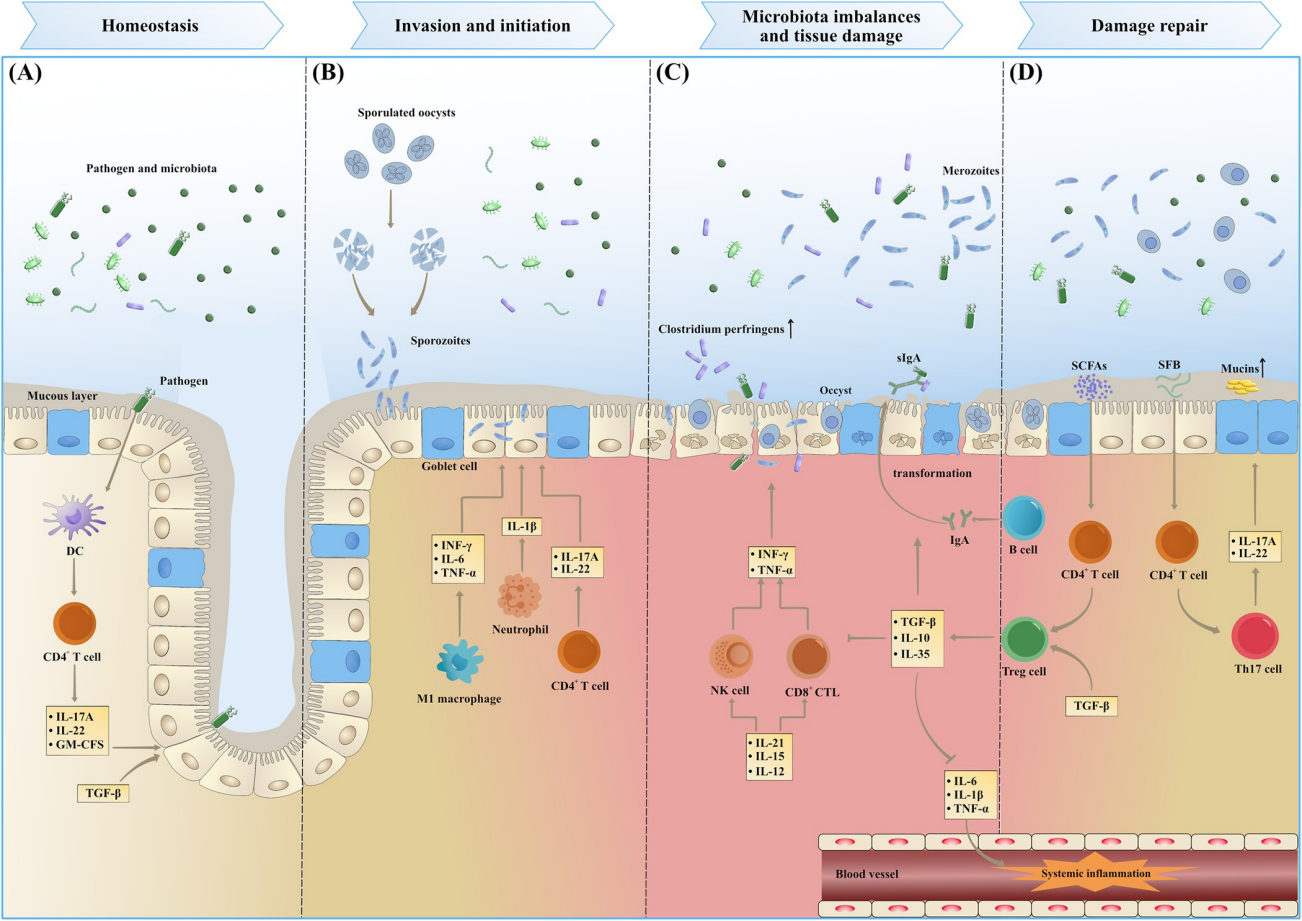
Intestinal microbiota in the pathogenesis of *Eimeria* infection. Intestinal microbiota experiencing orchestral arrangements at different stages of coccidiosis progression. (A) Homeostasis. Interleukin-22 (IL-22), IL-17A, and GM-CFS expressed by CD4^+^ T cells and ILCs in response to DCs present a good mucosal barrier for defense. (B) Invasion and initiation. Sporulated oocyst rupture and release sporozoites under the action of mechanical disruption and digestive enzymes. TNF-α, interferon-γ (IFN-γ), and IL-6 expressed by M1 macrophage play a significant role in inhibiting early invasion of sporozoites. IL-1β released from recruited neutrophils causes resistance to coccidia within the IECs. (C) Microbiota imbalances and tissue damage. Cytokines such as IL-15, IL-12, and IL-21 potentiate cytotoxic responses by CD8^+^ T cells and NK cells, which kills pathogens such as coccidia while exacerbating tissue damage. Microbiota imbalances lead to an increase in pathogenic bacteria, such as *Clostridium perfringens*, and causes host immune cells to secrete more pro-inflammatory cytokines, like IL-6, IL-15, and TNF-α. Subsequently, pro-inflammatory cytokines enter the blood vessel and trigger systemic inflammation in the host. (D) Damage repair. In the later stages of infection, overcompensated beneficial bacteria release more metabolites, such as SCFAs, in order to restore the balance of the intestinal microecosystem. *Segmented Filamentous Bacteria* (*SFB*) and SCFAs promote the differentiation of CD4^+^ T cells into Treg cells and Th17 cells, releasing more TGF-β, IL-10, and IL-35, which in turn inhibits excessive inflammatory responses and strengthens the intestinal mucosal barrier. GM-CFS, granulocyte–macrophage colony-stimulating factor; ILCs, innate lymphoid cells; DCs, dendritic cells; TNF-α, tumor necrosis factor-α; IECs, intestinal epithelial cells; NK cells, natural killer cells; SCFAs, short-chain fatty acids; Treg cells, regulatory T cells; TGF-β, transforming growth factor-β.

The diverse microbiome in the intestine plays a crucial role in digestion and protection of the gut (Fig 3A). Chickens with high intestinal microbial diversity are more stable and healthier than those with low diversity [105]. The intestinal microbiota, as a host symbiont, can even regulate species interactions, and changes in their communities have also been identified as early indicators of parasite infection [106]. There are 2 main possibilities for *Eimeria*–microbiota interactions. The first possibility is the direct role of the gut microbiota, which includes initiating the host’s intestinal immunity against *Eimeria* and influencing microbial diversity. The second is the indirect effect of intestinal microbiota, where their metabolites impact the invasion and colonization of *Eimeria*.

### Effects of the intestinal microbiota

The disturbance of the intestinal environment caused by *Eimeria* infection can result in a disorder of the intestinal flora known as dysbiosis [107,108]. This microenvironment creates favorable conditions for the growth of pathogenic bacteria, particularly *Clostridium*, which can lead to mild cases of diarrhea, severe cases of bloody stools, and even death. Specifically, *Clostridium perfringens* (*C*. *perfringens*) is the primary pathogen causing necrotizing enteritis (NE) in chickens [107,108]. *Eimeria* infection, particularly *E*. *maxima* infection, stimulates the production of intestinal mucus by inducing localized T-cell-mediated inflammatory responses, thereby providing a nutrient-rich environment for the growth of *C*. *perfringens*. [107]. Broilers coinfected with *E*. *maxima* and *C*. *perfringens* often experience more severe NE [109]. Furthermore, intestinal epithelial damage induced by coccidiosis exposes specific collagens within the extracellular matrix, which serve as binding sites for *C*. *perfringens*, leading to the production of potent toxins, such as necrotic enteritis B-like toxin (NetB) [110–112]. NetB, a pore-forming toxin, possesses the capability to lyse IECs, which serves as the direct cause of NE [113] (Fig 3B).

Multiple studies have supported the idea that beneficial bacteria in the gut can enhance the integrity of the intestinal barrier and prevent the invasion and colonization of *Eimeria* in IECs [114]. Intestinal segmented filamentous bacteria (SFB) can directly stimulate the differentiation of Th17 cells in the intestinal lamina propria, leading to increased production of IL-17 and IL-22 and strengthened mucosal immunity [115]. Lactic acid bacteria, an important component of the intestinal microbiota, can resist harmful bacterial colonization, regulate inflammatory responses, and protect the intestinal barrier [116]. *Roseburia*, another beneficial symbiotic bacterium in the intestinal flora, stimulates the secretion of immune cytokines by immune cells in the intestinal lamina propria, ultimately promoting the activation of regulatory T (Treg) cells, maintaining intestinal immunity, and improving disease resistance [117]. However, studies have indicated that infection with *E*. *tenella* reduces the abundance of *Lactobacillus* and *Roseburia* but increases the abundance of *Prevotella pectinovora* [118]. Combining with the transcriptome analysis of the host, *Prevotella pectinovora* has a significant positive correlation with fatty acid binding protein 1 (FABP1) [118]. Moreover, the down-regulation of FABP1 gene expression in poultry infected with *E*. *maxima* leads to the reduction of fatty acid utilization and intestinal immunity. [119,120]. Therefore, after *E*. *maxima* infection, *Prevotella pectinovora* and FABP1 may cooperatively function in host immunity and metabolic processes [118]. Additionally, during periods of polyinfection with several *Eimeria* species, including *E*. *acervulina*, *E*. *maxima*, and *E*. *tenella*, the average abundance of *lactobacilli* in the cecal microbiota decreases, and the abundance of pathogenic bacteria such as *Clostridium* and *Streptococcus* increases in chickens [121]. Similarly, our previous study revealed significant changes in the composition of the cecal microbiota in chickens infected with *E*. *tenella*, suggesting that such alterations may impact intestinal tissue and homeostasis [122]. Furthermore, evidence suggests that *Eimeria* infection reduces host resistance to disease by affecting the abundance and diversity of the intestinal microbiota [123] (Fig 3C). Therefore, strategies aimed at regulating the composition of the microbiome and its metabolism can help inhibit parasite replication or stimulate immune responses.

### Effect of microbial metabolites on *Eimeria* invasion

Microbial metabolites in the intestine play a crucial role in repairing damage and enhancing the immune resistance of the host during *Eimeria* challenge. Bile acids, synthesized from cholesterol in the liver, are secreted in the gut to facilitate lipid absorption [124]. However, the presence of the gut microbiota leads to the conversion of primary bile acids into unbound bile acids and secondary bile acids through dissociation and dihydroxylation reactions. These microbial bile acid metabolites are capable of controlling host immunological homeostasis and inhibiting intestinal inflammation, thereby protecting the intestinal barrier [125]. During NE infection caused by *E*. *maxima* and *C*. *perfringens*, bile acid secretion is reduced, but the intestinal bacterial metabolism of secondary bile acids can reduce the NE-induced inflammatory response and the colonization of *C*. *perfringens* [126]. Although direct research on the role of secondary bile acids in *Eimeria* infections is lacking, they have been proven to significantly inhibit the growth of *Cryptosporidium parvum* (*C*. *parvum*) [127]. Additionally, oral administration of alpha-linolenic acid (ALA), a type of short-chain fatty acid (SCFA), has been shown to inhibit the MyD88/NF-κB pathway and reduce colitis induced by *T*. *gondii* [128]. The concentration of SCFAs fluctuates during *Eimeria* spp. infection, initially showing a significant decrease, followed by a sharp but brief increase, and finally returning to the noninfected level of the host. This fluctuation is associated with changes in the intestinal microbiota [129,130]. It has been proposed that the transient increase in SCFAs may be a result of the overcompensation of beneficial bacteria to restore the balance of the intestinal microecosystem [131].

Intestinal microbiota participates in the host tryptophan metabolism by generating metabolites such as indole, which activate the aryl hydrocarbon receptor (AhR) to stimulate the immune system and enhance the intestinal epithelial barrier [132]. Indole-3-carboxylate (ICCOH) regulates the expression of TJs to enhance intestinal barrier integrity during *E*. *maxima* challenge [133]. ICCOH reduces the expression of IL-1β, IFN-γ, and IL-10 through the regulation of Th cells, thereby mitigating the damaging effects of inflammatory cytokine infiltration on TJs [100–102]. Dietary supplements such as 3,3′-diindolemethane (DIM) and indole-3-methanol (I3C) have also shown potential for alleviating intestinal tissue damage by modulating T-cell immunity [134]. Dietary supplementation with AhR pro-ligand I3C activates CD8^+^ intraepithelial lymphocytes (IELs) in the intestine, conferring resistance against the growth of *Cryptosporidium tyzzeri* [135]. Moreover, an in vitro study has demonstrated that indole can directly reduce the membrane potential in the parasite mitosome, a degenerate mitochondrion of *Cryptosporidium*, subsequently resulting in developmental delay of the parasite. The growth restriction of *C*. *parvum* by indole acts directly on the parasite, independent of the host AhR pathway [127].

In summary, microbial products play important roles during *Eimeria* challenge by exerting anti-inflammatory effects, regulating immune function, and improving the integrity of the intestinal barrier (Fig 3D).

### Intestinal microbes appear to aid *Eimeria* invasion

It appears that the invasion of IECs by *Eimeria* further disrupts the balance of the intestinal microbiota, leading to severe harm to the host. Previous studies have focused on the negative effects caused by the disturbance of intestinal microbial stability during parasite invasion [121]. However, an interesting study using a sterile chicken model of infection showed that the absence of microbiota also affected the development of *E*. *tenella* in the intestine. *E*. *tenella* oocysts developed more slowly in sterile chickens than in conventional chickens [94]. Additionally, Gaboriaud [136] discovered that, compared to sterile chickens, the cecal microbiota accelerated the promotion of the acute inflammatory response and the loss of intestinal barrier integrity during *E*. *tenella* infection in conventional chickens. This finding suggested that the microbiota plays a role in facilitating *Eimeria* transmission among IECs. Similar studies have shown that interactions between the microbiota and host immune system can promote pathogen infection by altering metabolism and the immune environment [137,138]. Although it is recognized that the microbiota can enhance the host immune capacity to resist pathogen invasion, changes in the microenvironment can contribute to pathogen invasion and may be associated with reduced immune capacity [139]. However, further in-depth exploration is required to fully understand this complex phenomenon.

## Interaction between *Eimeria* and the intestinal immune barrier

Comparative genomic analysis between *Cryptosporidium* chipmunk genotype I, a novel zoonotic pathogen in humans, and *C*. *parvum* revealed positive selection in genes encoding invasion-related proteins, secreted proteins, and surface-associated proteins that may be involved in host immune responses [140]. Additionally, comparative analysis of the genomes of *C*. *parvum* and *C*. *hominis* also revealed that the genes of both species show strong positive selection, which encode proteins that interact with the host immune response [141]. These findings suggest that selective pressures exerted by host immune responses play a crucial role in shaping the patterns of genetic diversity in parasite genomes. Generally, immune cells control infection by engulfing and degrading parasites, but certain parasites, such as *T*. *gondii* and *Eimeria*, can evade this defense mechanism and multiply within host cells, partly due to their unique virulence factors [142,143].

### Immune evasion of *Eimeria*

**Fig 4:**
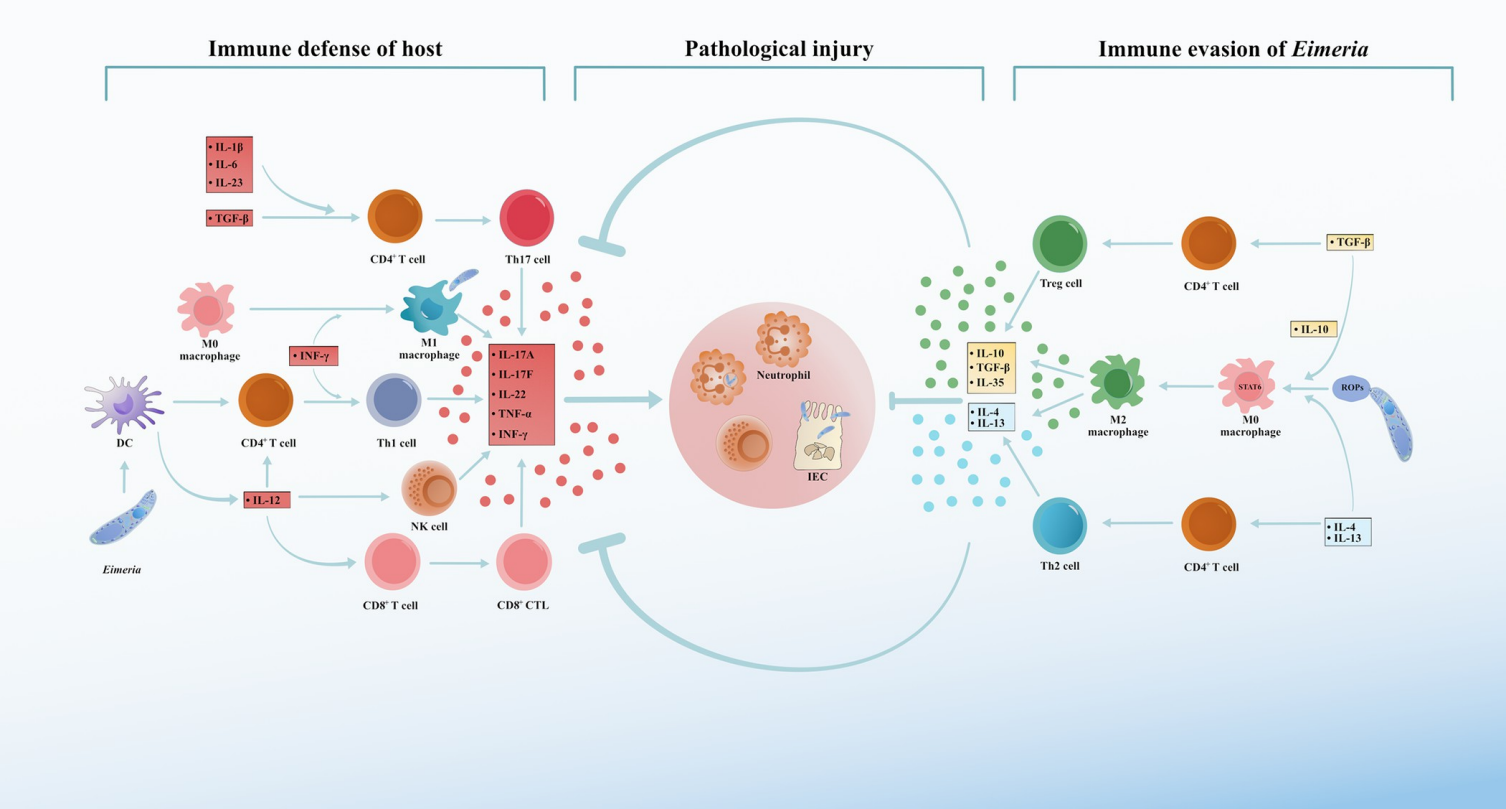
Interactions between immune evasion of *Eimeria* with immune defense of host. During the early stages of *Eimeria* invasion, DCs are the first to respond by releasing interleukin-12 (IL-12). IL-12 stimulates the differentiation of CD4^+^ T cells into Th1 cells, which in turn produce TNF-α and interferon-γ (IFN-γ), activating the immune system to resist the invasion. Additionally, IL-1β, IL-6, IL-23, and TGF-β can promote the differentiation of CD4^+^ T cells into Th17 cells. IL-17A, IL-17F, and TNF-α produced by Th17 cells, and IFN-γ produced by Th1 cells and NK cells recruit and/or activate neutrophils and macrophages jointly resist *Eimeria* invasion. IL-17 and IL-22 also protect intestinal epithelial barrier. These inflammatory responses promote killing of *Eimeria* by M1 macrophages and CD8^+^ cytotoxic T lymphocytes (CTLs), but also lead to tissue damage. *Eimeria* learns to exploit host’s own immune regulation for their own evasion during a long struggle with the host. The ROPs secreted by *Eimeria* stimulate the polarization of M0 macrophages to M2 macrophages, which in turn secrete more IL-4, IL-13, IL-10, IL-35, and TGF-β. These cytokines are capable of directly or indirectly inhibiting host killing of *Eimeria*. TGF-β further stimulates the polarization of macrophages towards the M2 phenotype through synergistic interactions with IL-10. Additionally, IL-4 and IL-13 promote the differentiation of CD4^+^ T cells into Th2 cells, while TGF-β stimulates the differentiation of CD4^+^ T cells into Treg cells. The further release of anti-inflammatory cytokines inhibits the host inflammatory response, aiding in the immune evasion of *Eimeria*. DCs, dendritic cells; TNF-α, tumor necrosis factor-α; TGF-β, transforming growth factor-β; Th17 cells, T helper 17 cells; NK cells, natural killer cells; ROPs, rhoptry bulb proteins; Treg cells, regulatory T cells.

As the primary component of the innate immune defense system, macrophages play a crucial role in the identification and phagocytosis of pathogens, including apicomplexan parasites. The activation of macrophages to produce distinct functional phenotypes in response to specific stimuli is known as polarization. A study utilizing a combination of image analysis and live-cell imaging demonstrated that the mechanisms of *T*. *gondii* invasion and survival appear to be altered in macrophages exposed to *E*. *tenella* [144]. The phosphorylation of STAT6 has been shown to induce M2 polarization in macrophages, whereas the Th2 cytokines IL-4 and IL-13 are capable of promoting the phosphorylation of STAT6 or the transcription of response genes [145]. As an intracellular parasite, *E*. *tenella* induces IL-4 expression in the host, potentially diverting immune responses away from anti-*Eimeria* Th1 and IFN-γ production and towards less effective Th2 phenotypes [146]. During *Eimeria* challenge, dietary peptide-specific antibodies against IL-4 have been found to modify systemic immune cell responses, thereby enhancing host resistance [147]. Similar findings have been observed in *T*. *gondii* infection experiments in vivo, where M2 polarization has been associated with the suppression of antiparasitic immune responses [148]. Furthermore, the newly identified effector protein *Tg*SOS1 in *T*. *gondii* has been shown to prolong ROP-initiated STAT6 signaling. Sustained STAT6 signaling is crucial for the M2 polarization of *T*. *gondii*-infected macrophages and the efficient formation of bradyzoite cysts in neurons [149]. ROP16, a rhoptry protein, exhibits a conserved predicted C-terminal domain across Apicomplexa, whereas the recognizable N-terminal domain is specific to coccidia, including *Eimeria* [149]. Although ROP16 and *Tg*SOS1 have not been fully characterized, available evidence suggests that similar immune evasion mechanisms induced by functional proteins with an N-terminal domain may exist in coccidia. Moreover, IL-10 is believed to promote intracellular parasite infection by disabling Th1 cell-type responses and/or deactivating parasitized tissue macrophages [150]. Oral antibodies targeting IL-10 have been found to reduce the decrease in growth rate caused by *Eimeria* spp. infection in broiler chickens [151]. IL-10, which was initially described in Th2 cells [152], is coexpressed with other Th2 cytokines, including IL-4, IL-5, and IL-13. Coexpression is regulated by Th2-associated signaling pathways and transcription factors such as IL-4, STAT6, and GATA3 [153–155]. During *Eimeria* infection, overpolarized M2 macrophages are capable of secreting higher levels of IL-10 and transforming growth factor-β (TGF-β) [156,157]. Additionally, Tregs can produce IL-10. Kim has also suggested that one of the potential mechanisms of immune evasion in *Eimeria* spp. is the stimulation of Tregs by the parasite to produce IL-10, thereby inhibiting IFN-γ-related Th1 immune responses and ultimately promoting parasite invasion and survival [158] (Fig 4).

IL-12 plays a significant role in inducing INF-γ by activating 2 pathways (Fig 4). First, it induces the differentiation of naïve T cells into Th1 cells, resulting in the secretion of INF-γ. Second, IL-12 promotes the secretion of more IFN-γ by NK cells and CD8^+^ cytotoxic T lymphocytes (CTLs) [159,160]. An in vitro study confirmed the inhibitory effect of 4 molecules derived from *E*. *maxima*, namely, *Em*SAG, *Em*HPSP-1, *Em*HPSP-2, and *Em*HPSP-3, on the expression of IFN-γ in chicken HD11 macrophages and bone marrow-derived dendritic cells (BMDCs). The underlying mechanism of action involves the inhibition of IL-12 secretion by up-regulating the expression of phosphorylated ERK1/2 proteins [88]. INF-γ effectively suppresses the development of *E*. *tenella* in the host and is negatively correlated with oocyst shedding [161,162]. This finding is consistent with observations in other apicomplexan parasites, where mice lacking INF-γ are more susceptible to *Cryptosporidium* infection and experience more severe symptoms than wild-type mice [163]. In addition, *T*. *gondii* strains are lethal to KO but not SW mice (INF-γ knockout [KO], Swiss Webster outbred [SW]) [164]. Consequently, bolstering Th2 immunity and inhibiting Th1 immunity has become an essential approach for evading the host immune response during *Eimeria* invasion. Tregs also contribute to this mechanism of immune evasion.

### Immune resistance of the host

The escape mechanism of *Eimeria* is not absolute. A novel independent protein, EtROP21, was identified in *E*. *tenella* and could contribute to the increase in both host IFN-γ and IL-2 (Th1-like cytokine) and IL-4 (Th2-like cytokine) levels and to a Th1/Th2-mixed immune response [165]. In vitro and in vivo experiments have shown that IFN-γ, a direct factor that induces macrophage M1-type polarization, can reduce *Eimeria* infection [166,167]. However, INF-γ expression was also found to be enhanced [165], which could be associated with host resistance to immune evasion. To elucidate the potential mechanisms involved, TGF-β was found to be highly expressed during *E*. *tenella* invasion [118], and the up-regulation of TGF-β also attracted unpolarized macrophages to polarize into the M2 state [168]. Similarly, in intestinal immunity, M2 macrophages are capable of producing more TGF-β, indicating the involvement of TGF-β in host immune evasion [169]. Previous research has demonstrated that TGF-β, a crucial immunomodulator, can inhibit Th1 cells, Th2 cells, and CTLs but can interact with other cytokines to induce the differentiation of Tregs and Th17 cells [170]. As a downstream molecule of TGF-β, together with IL-10 and IL-21, it can enhance the conversion of IgA to secretory IgA (sIgA) [171]. Our prior investigations confirmed that the invasion of *E*. *tenella* disrupts the intestinal mucosal barrier, and the protection of the intestinal mucosa by sIgA is particularly important [172]. Furthermore, TGF-β also regulates the expression of adhesion molecules, prevents goblet cell depletion and dysbiosis, and enhances TJs [173]. These findings suggest that TGF-β plays a dual role in mediating host immunity, which may be associated with its role in maintaining immune homeostasis. Therefore, further in-depth examination of the role of TGF-β in the immune response induced by intracellular parasites is warranted.

IL-17 plays a significant role in the immune response to *Eimeria* infection. High levels of TGF-β promote the differentiation of CD4^+^ T cells into Th17 cells, with IL-1β, IL-6, and IL-23 acting in combination [174]. Th17 cells exhibit plasticity in cytokine production in vivo, shifting from primary IL-17 production to primary IFN-γ production in response to specific environmental stimuli [151]. Furthermore, IL-17, which is typically produced by Th17 cells, plays a crucial role in host resistance to *Eimeria* infection. One study demonstrated that immunization with recombinant *E*. *magna* GAM56 (r*Em*GAM56) and ROP17 (r*Em*ROP17) proteins significantly increased the levels of INF-γ, IL-2, and IL-17 in the host [175]. In a previous study, we found that the mRNA expression level of IL-17 in the host was significantly greater following *E*. *tenella* infection [176]. However, contradictory findings have been reported, indicating the down-regulation of IL-17A expression in the cecum after primary infection with *E*. *tenella* [177]. Compared with chickens infected with *C*. *perfringens* alone, chickens coinfected with *E*. *maxima* and *C*. *perfringens* also exhibited decreased intestinal IL-17A transcript levels. This finding suggested that the expression of IL-17A in the gut is dependent on the infecting *Eimeria* species and the time postinfection. Therefore, promoting the immune regulation strategy of IL-17 could enhance immune protection against *Eimeria* to some extent. These findings suggest that IL-17 may play a role in the pathogenesis of *E*. *tenella* infection.

Similar studies have reported the involvement of IL-17 in other intracellular parasites. IL-17 has a protective role against *Trypanosoma cruzi* in mice by controlling the expression of IL-10 through limited neutrophil recruitment. This indicates that the balance between IL-17 and IL-10 can determine the result of infection [178]. IL-17 can also promote protection against *T*. *gondii* by recruiting neutrophils [179]. The DNA vaccine encoding ROP13 induces IL-17 production in mice, which exhibits a protective effect against *T*. *gondii* infection [180]. Previous studies have shown that hosts primarily eliminate intracellular *T*. *gondii* by activating Th1 immunity to produce cytokines such as IFN-γ, IL-12, and TNF-α [181–183]. Intriguingly, during *Trypanosoma cruzi* infection, host Th17 cells are more protective than Th1 cells [184]. This may be attributed to the role of IL-17 in enhancing the immunity of infected macrophages [185]. Th17 cells effectively promote a robust CD8^+^ T cell response that is independent of IL-17 [184]. Similarly, CD4^+^ and CD8^+^ T lymphocytes expressing IL-17 may play a crucial role in the inflammatory response induced by *T*. *gondii*, thereby aiding in the control of parasite invasion and replication [186]. Additionally, mice deficient in IL-23 (p19KO) and IL-17 (17KO) have greater parasitaemia and earlier mortality during *Plasmodium berghei* infection than wild-type (WT) controls. Collectively, these studies suggest that, whereas IL-17 has a protective effect against intracellular parasites, it can also mediate immunopathology in some cases [187].

Furthermore, IL-10 has been shown to directly restrain proinflammatory Th17 and “Th1 plus Th17” cells, which are positive for IL-17A and INF-γ. This is achieved by targeting the functional IL-10 receptor [168]. Therefore, it is reasonable to hypothesize that the expression levels of IL-17 are the result of competition between *Eimeria* and the host (Fig 4). The activation or inhibition of Th17 cells may serve as a “hot” battlefield where intracellular parasites compete with the host for immune control.

## Concluding remarks

In recent years, considerable attention has been paid to the mechanisms of parasite–host interactions. Through clinical research studies, it was found that *Eimeria* exhibits strong host and tissue tropism, which could be driven by a combination of microneme specificity and host receptor specificity. During the early stages of infection, *Eimeria* initiates invasion by releasing MICs, particularly MIC3 and MIC8, which specifically recognize and adhere to surface molecules on host IECs. Additionally, we have predicted the invasion process of *Eimeria* based on comparisons with studies on other apicomplexan parasites. Although multiple studies indicated that most of the MJ complex proteins are conserved among Apicomplexa, further verification of the structure of AMAs-RONs complexes in *Eimeria* species will be beneficial for the biology and vaccine development of *Eimeria*. The molecules released by *Eimeria* disrupt the TJs of IECs through interactions with surface molecules on host cells, thereby accelerating invasion and colonization. During the later stages of infection, sporozoites/merozoites already colonized within IECs perpetuate the disruption of tight junctions by promoting intestinal inflammatory responses, thereby facilitating continued invasion. This demonstrated the cunning nature of *Eimeria* in achieving parasitism.

Previous studies have focused on the negative effects of disturbances in intestinal microbial stability during parasite invasion on the host. It is noteworthy that recent studies have shown that interactions between the microbiota and host immune system can promote pathogen infection by altering metabolism and the immune environment. Although it is recognized that microbiota can enhance the host immune capacity to resist *Eimeria* invasion, changes in the microenvironment can, in turn, contribute to a more severe invasion of *Eimeria*. In the future, leveraging fecal microbiota transplantation (FMT) and microbiome studies to investigate the influence of specific bacteria or microbial communities, with a focus not on the host but on the parasites, may hold the key to unraveling this phenomenon.

Apicomplexan parasites, through their intricate and sophisticated immune evasion mechanisms, successfully evade host immune defense. This biological phenomenon has posed a significant challenge in the field of biomedical research for an extended period. We also discussed the immune evasion mechanisms of *Eimeria*, which may be related to the programming and migration of macrophages. It has been proposed that migratory activation of macrophages may facilitate the dissemination of intracellular parasites, which could potentially lead to the inefficacy of cell therapies [142]. Furthermore, we have emphasized that the host employs sophisticated immune regulatory mechanisms to suppress immune evasion by parasites, thereby sustaining a balanced immune response. Recently, a single-cell sequencing study on *Plasmodium* found that abortive cells exhibited a distinct gene expression signature enriched in immune recruitment genes, such as the IFN-γ response, compared to infected cells [188]. Therefore, considering these factors, targeted immunotherapy may represent a promising option in the future.

Our literature review has indicated that *Eimeria* undergoes a series of changes during invasion, colonization, and development. These processes require dynamic response mechanisms that adapt to immune evasion strategies, as well as other host-specific and stage-specific interactions. However, compared to those of other apicomplexan parasites, the mechanisms underlying *Eimeria*–host interactions are poorly understood. Although studies on *Eimeria* can utilize existing information from other apicomplexan parasites, it is important to note that *Eimeria* exhibits different pathogenic mechanisms and behaviors. Fortunately, advancements in technologies and bioinformatics have started to unravel the secrets of parasite–host interactions in a systematic manner. For instance, comparative genome screening between hosts and parasites, as well as single-cell sequencing studies across different stages, have revealed numerous novel mechanisms of parasite–host interactions. Nevertheless, the complex regulatory processes within the host still render the specific molecular mechanisms elusive (see **Outstanding questions**). Identifying the mechanisms governing *Eimeria*–host interactions will pave the way for developing new therapeutic approaches to combat the challenges posed by *Eimeria* in animal husbandry and public health.

**Box 1. Influence of environmental factors in the host intestinal tract on *Eimeria***

Sporulated oocysts can initiate replication when orally ingested by a host [4]. In this scenario, sporulated oocysts rupture and release sporozoites due to mechanical disruption and digestive enzymes. The release of sporozoites may also be influenced by carbon dioxide (CO_2_), temperature, and digestive enzymes. Research has demonstrated the critical role of host temperature in the release of sporozoites [189–192]. Additionally, *Eimeria* oocysts treated with CO_2_ have been observed to be more fragile than those without treatment [193]. Consequently, oocysts exposed to CO_2_ in crop regions are likely to be more susceptible to breakage or alteration of the gizzard. Similarly, a study showed that with appropriate exposure to CO_2_ and temperature, the oocyst wall of *E*. *tenella* becomes more permeable or susceptible to trypsin and bile in vitro. Trypsin can digest the oocyst wall, resulting in thinning and rupture [194]. This finding was confirmed through the use of electron microscopy and fluorescent dyes in vitro [195,196]. However, it is important to note that sporozoite excystation occurs after the breakdown of sporocysts [194,197]. Trypsin or chymotrypsin digests the stieda body, creating a hole in the sporocyst membrane and facilitating sporozoite exposure. Although bile is not necessary, its absence slows the release and movement of sporozoites. Bile not only promotes the entry of digestive enzymes into sporocysts by altering microtubules but also affects the lipoproteins of the stieda body within *Eimeria* oocysts [198,199]. Sodium taurocholate (NaTC) enhances the protein secretion and gliding motility of *Cryptosporidium* sporozoites, which is crucial for successful parasite invasion [192]. In chickens, trypsin and bile are emptied through a duct on a common nipple in the duodenal wall [195]. This physiological condition facilitates the release of sporozoites in the host intestine through a combination of bile, pancreatic enzymes, and other unidentified factors [199,200]. Furthermore, this specific intestinal environment also contributes to further tissue tropism for *Eimeria* development.

**Outstanding questions**

1. Which endo-environmental factors and receptors in host plays a key role in *Eimeria* invasion?
2. Does the immune evasion mechanism of *Eimeria* change in the different parasite stages? What are the host countermeasures against immune evasion by *Eimeria*?
3. Which of the gut microbes inhibit *Eimeria* invasion and colonization, and which promote parasites invasion? How do differences in gut microbiota and microbial metabolites determine the outcome of infection?
4. What is the role of Treg cells and Th17 cells in the immune arms race between *Eimeria* with host?
